# Neurodevelopment of children exposed in utero to treatment of maternal malignancy

**DOI:** 10.1054/bjoc.2001.2090

**Published:** 2001-11

**Authors:** I Nulman, D Laslo, S Fried, E Uleryk, M Lishner, G Koren

**Affiliations:** Motherisk Program Division of Clinical Pharmacology/Toxicology, Department of Pediatrics, The Hospital for Sick Children, University of Toronto, Toronto, Canada

**Keywords:** malignancy, child development, antineoplastic medications, radiation, glucocorticoids, bromocriptine

## Abstract

Cancer is the second most common cause of death during the reproductive years, complicating approximately 1/1000 pregnancies. The occurrence of cancer during gestation is likely to increase as a result of a woman's tendency to delay childbearing. Improved diagnostic techniques for malignancies increases detection of cancer during pregnancy. Malignant conditions during gestation are believed to be associated with an increase in poor perinatal and fetal outcomes that are often due to maternal treatment. Physicians should weigh the benefits of treatment against the risks of fetal exposure. To date, most reports have focused on morphologic observations made very close to the time of delivery with little data collected on children's long-term neurodevelopment following in utero exposure to malignancy and treatment. Because the brain differentiates throughout pregnancy and in early postnatal life, damage may occur even after first trimester exposure. The possible delayed effects of treatment on a child's neurological, intellectual and behavioural functioning have never been systematically evaluated. The goal of this report was to summarize all related issues into one review to facilitate both practitioners' and patients' access to known data on fetal risks and safety. © 2001 Cancer Research Campaign http://www.bjcancer.com


*British Journal of Cancer* (2001) **85**(11), 1611–1618
© 2001 Cancer Research Campaign
doi: 10.1054/ bjoc.2001.2090, available online at http://www.idealibrary.com on IDEAL

Review

### Neurodevelopment of children exposed in utero to treatment of maternal malignancy

**I Nulman[1,2,3,4], D Laslo[1], S Fried[4], E Uleryk[3], M Lishner[1] and G Koren[1,2,3,4]**

[1]Motherisk Program Division of Clinical Pharmacology/Toxicology; [2]Department of Pediatrics; [3]The Hospital for Sick Children and [4]University of Toronto, Toronto, Canada

**Summary** Cancer is the second most common cause of death during the reproductive years, complicating approximately 1/1000 pregnancies. The occurrence of cancer during gestation is likely to increase as a result of a woman's tendency to delay childbearing. Improved diagnostic techniques for malignancies increases detection of cancer during pregnancy. Malignant conditions during gestation are believed to be associated with an increase in poor perinatal and fetal outcomes that are often due to maternal treatment. Physicians should weigh the benefits of treatment against the risks of fetal exposure. To date, most reports have focused on morphologic observations made very close to the time of delivery with little data collected on children's long-term neurodevelopment following in utero exposure to malignancy and treatment. Because the brain differentiates throughout pregnancy and in early postnatal life, damage may occur even after first trimester exposure. The possible delayed effects of treatment on a child's neurological, intellectual and behavioural functioning have never been systematically evaluated. The goal of this report was to summarize all related issues into one review to facilitate both practitioners' and patients' access to known data on fetal risks and safety. © 2001 Cancer Research Campaign http://www.bjcancer.com

**Keywords**: malignancy; child development; antineoplastic medications; radiation; glucocorticoids; bromocriptine

### METHODS

The data presented in this paper are based on a Medline search of the literature from 1966 to May 2001 and a Cancerlit search from 1983 to May 2001. Both searches were performed on Ovid Medline. The search strategy combined the following four concepts using available MeSH terms:

Concept 1 (exp pregnancy or exp pregnancy complication); and

Concept 2 (exp developmental disabilites or exp 'behaviour and behaviour mechanisms' or exp cognition disorders); and

Concept 3 (exp antibiotics, anthracycline or exp antibiotics, antineoplastic, exp antineoplastic agents, or antineoplastic agents, combined or exp antineoplastic agents, hormonal or exp antineoplastic and immunosuppressive agents or exp radioisotopes or exp anesthetics or prednisone or bromocriptine or glucocorticoids or exp estrogens); and

Concept 4 (exp neoplasms).

The search was not limited to specific languages, age groups or human studies. The studies were reviewed for details on fetal brain development evidenced postnatally by different physical, neurological and neurodevelopmental tests.

### MATERNAL CANCER

Whether an association of maternal cancer with pregnancy has an undesirable effect on neurodevelopment in later childhood remains largely unknown. Metastasis to the placenta and/or fetus is very rare (Gililland and Weinstein, 1983; Potter, 1970). Melanoma is the most frequent of the few malignancies that do metastasize to the placenta and fetus (Gililland et al, 1983). Live born children exposed to such malignancies tend to die within a 1 day to 10 month period. Comprehensive reports about physical and mental health of the surviving children are not available.

The cancer patient has an increased tendency to experience febrile illnesses due to infections and/or as a result of the tumour itself. The relationship between hyperthermia, fetal brain development, and the incidence of impairment in human children has not been fully addressed. However, many human studies do support the hypothesis that maternal fever in early pregnancy is associated with an increased risk of NTDs and microphthalmia (Chambers et al, 1998; Miller et al, 1978; Layde et al, 1980; Clarren et al, 1979).

Malignancies may also be associated with maternal malnutrition and adverse neonatal outcome (Metcoff et al, 1981; Brown et al, 1981). Although animal studies have shown that severe maternal undernutrition can result in stillbirths and increased perinatal mortality, retrospective analyses of human data obtained during historical periods of starvation have shown little or no adverse fetal effects (Pritchard et al, 1985). Several studies have suggested that severe ketoacidosis due to dehydration or severe weight loss may pose an additional risk to the developing fetus (Naeye et al, 1981). On the other hand, analysis of males at age 19 born to mothers starved during pregnancy failed to document any differences in IQ score between these boys and the general male population (Stein et al, 1972).

### SURGERY

Surgical interventions may be required for the evaluation and treatment of malignancies diagnosed in pregnancy. Each year about 0.5% to 2% of pregnant women in North America undergo

*Received 18 July 2001*
*Accepted 2 August 2001*

*Correspondence to:* I Nulman, The Motherisk Program, Division of Clinical Pharmacology, The Hospital for Sick Children, 555 University Avenue, Toronto, Ontario M5G 1X8, Canada surgery for reasons unrelated to their pregnancy and approximately 7000 to 30 000 of these women are in the first trimester of pregnancy (Sylvester et al, 1994), a critical time for the development of the central nervous system (CNS) (Webster et al, 1988). This is also the time period when the woman may not be aware of her pregnancy. Some women are occupationally exposed to anaesthetics throughout gestation. Little is known about neurodevelopmental outcomes due to exposure in either surgical or occupational conditions. During surgery the fetus is exposed to a potential risk from the effects of anaesthetic agents in addition to complications that may arise during or as a result of maternal surgery. Surgery in pregnancy is often associated with hypotension, hypoxia, coagulation, metabolic disturbances, and decreased utero-placental perfusion secondary to prolonged maintenance in the supine position. Each of these conditions can threaten fetal well being (Doll et al, 1988).

There are studies that establish the safety of the most commonly used anaesthetic agents during pregnancy, including nitrous oxide, enflurane, barbiturates, and narcotics (Schardein, 1985). Friedman (1988) reviewed possible teratogenic effects of general anaesthetics, local anaesthetics, and occupational exposure to anaesthetic gases. He concluded that data available on these agents do not suggest a major teratogenic risk in humans.

Kallen and Mazze (1990), in a case control study, assessed the pregnancy outcome of 2252 infants born to women who had surgery in the first trimester. Six neonates (expected number = 2.5) had a definite diagnosis of neural tube defect (NTD). Five of these 6 mothers were operated on in the fourth and fifth weeks of gestation, the window of exposure in which NTDs may take place. Sylvester (1994), in a large population-based case-controlled study, evaluated whether exposure to maternal general anaesthesia is associated with an increase in fetal CNS abnormalities. Twelve of 694 mothers with infants who had CNS defects reported having first trimester surgery with anaesthesia. Thirty-four of the 2984 controls were exposed to general anaesthesia at the same time of gestation (OR = 1.7, 95% CI-0.8,3.3). The strongest association was observed between exposure to anaesthesia and hydrocephalus, as well as eye defects. Although available studies have indicated that the CNS is the most sensitive organ to surgical and anaesthetic damage, none of the studies have addressed later childhood neurodevelopment or cognition. Functional CNS abnormalities may exist even when structural abnormalities are not observed.

### MEDICATIONS

### Cytotoxic drugs

The common denominator of anti-cancer drugs is the ability to affect cell division adversely. Therefore, the same qualities that make those compounds desirable for cancer therapy, may also render them detrimental to the developing embryo. The dose of medication and time of exposure are critical during embryogenesis when susceptibility to teratogenic agents is high (Webster et al, 1988). A number of studies focused on congenital malformations at the time of delivery, and concluded that when chemotherapy is administered to women before conception or after the first trimester, in the majority of cases normal births are experienced (Harada, 1978; Gililland and Weinstein, 1983; Doll et al, 1988; Cantini and Yanes, 1984). These conclusions may not apply to the CNS, which develops throughout gestation and postnatally. Xenobiotics, including some heavy metals, ethanol and cocaine, are known to adversely affect CNS development during the second and third trimester (Koren et al, 1994; Harada, 1978).

Cognitive and behavioural functioning of children exposed in utero to chemotherapy at different periods of gestation remain largely undefined. Available data are based on small series and case reports. These reports suggest that gross and mental development of children exposed in utero to chemotherapy appears to be normal, but most authors did not conduct formal motor, cognitive and behavioural tests. As a result, they may have missed the opportunity to detect more subtle neurodevelopmental abnormalities. In case reports of women who were treated with a variety of chemotherapeutic agents and the developmental outcome was reported, the infants presented normal growth and developmental milestones at 3–21 months following delivery (Odom et al, 1990). However, Reynoso (1987) and Cantini (1984) reported 2 children with mental and or growth retardation born to mothers treated in pregnancy for acute leukaemia.

Presented below are the data concerning late effects of chemotherapy on children's neurodevelopment (Table 1). Blatt et al (1980) assessed retrospectively pregnancy outcome in patients who received aggressive moderate to high-dose combination chemotherapy for various oncologic diseases. Two patients conceived during treatment, which was continued for the first 2 months of gestation, and two other women started chemotherapy in the second and third trimester respectively. At the time of evaluation, the offspring ranged in age from several days to 12 years. Development was evaluated using the Denver Developmental Screening Test, and school performance was ascertained by history. Growth and development as well as school performance appeared to be normal in these children.

The peak incidence of Hodgkin's disease occurs during the reproductive years. Therefore, it is not surprising that there have been more reports on long-term follow-up for Hodgkin's disease than for other malignancies. Baisogolov and Shishkin (1985) reported on the pregnancy outcome of 78 retrospectively collected patients with Hodgkin's disease who delivered 89 children. The data were collected from the parents by local paediatricians. Twenty-one women conceived while undergoing chemotherapy. The psychomotor development of the case children was not different from that of the controls (children of healthy mothers). Seventeen out of 19 school-aged children were considered as 'good' or 'excellent' students. Twelve of these children were good in mathematics, 6 were in advanced programs to study foreign languages, 4 studied music, and 10 were good in sports. Another 7 children were gifted in other areas. A study conducted in Poland reported on the outcome of 20 pregnancies in 16 women with Hodgkin's disease (Balcewicz-Sablinska et al, 1990). In three pregnancies, cytostatic treatment was given after the first trimester. The course and labour of the observed pregnancies were normal. All babies were born healthy and were followed for a period of 6 years. The development of these children was reported to be normal, although formal psychological assessments were not performed.

The pregnancy outcome of women with haematologic malignancies, screened between 1970 and 1986, was presented by Aviles et al (1991). Forty-three children were born to 43 mothers who were treated with chemotherapy for non-Hodgkin's lymphoma, Hodgkin's disease, acute leukaemia, or chronic granulocytic leukaemia. Nineteen women received chemotherapy during the first trimester. The children's ages ranged from 3 to 19 years in 1989 at the time of testing. They were evaluated by

 *© 2001 Cancer Research Campaign*

© 2001 Cancer Research Campaign

**Table 1** In utero exposure to chemotherapeutic agents

| Indication | Authors | Time of exposure in pregnancy | Study design | Sample size *n* = 111 | Age assessed | Medications | Tests | Results |
|---|---|---|---|---|---|---|---|---|
| Different forms of malignancies | Blatt et al, 1980 | Preconceptionally or 1st trimester | Retrospective | 4 | 1 month to 12 years old | Combined chemotherapy | Denver Developmental Screening Test. School reports | Normal development and school performance |
| Hodgkin's disease | Baisogolov and Shishkin, 1985 | – | Retrospective | 19 | 1 to 14 years old | Combined chemotherapy | Parent and school reports | Normal development |
| Hodgkin's disease | Balcewicz-Sablinska et al, 1990 | 1st trimester | Retrospective | 3 | Up to age 6 | MOPP | No formal testing | Normal development |
| Hodgkin's disease | Aviles et al, 1991 | 1st and 2nd trimesters | Retrospective, controlled | 15 | 3 to 17 years | MOPP, ABVD | Wechsler and Bender-Gestalt cognitive tests. School report | Not different from controls |
| Haematological malignacies | Aviles et al, 1991 | 1st trimester, 2nd and 3rd trimester | Retrospective, controlled | 43 | 3 to 19 years | Combination chemotherapy | Wechsler and Bender-Gestalt cognitive tests. School report | Not different from controls |
| Acute leukaemia | Aviles et al, 1988 | 1st trimester or sometime during pregnancy | Retrospective, controlled | 17 | 4 to 22 years | Combination chemotherapy | Wechsler and Bender-Gestalt cognitive tests. School reports | Not different from controls |
| Rheumatic disease | Kozlowsky et al, 1990 | 1st trimester | Retrospective | 5 | 3.7 to 16.7 years | Low-dose methotrexate | Parent reports | Normal development |
| Occupational exposure | Medkova 1991 | Preconceptionally and/or during pregnancy | Retrospective, controlled | 5 | – | Low dose cytostatics | No formal testing | Normal development |

physical and neurological examination in a 'blinded' manner. Evaluation of the children's school performance was obtained from their teachers. The Wechsler and Bender-Gestalt tests were administered to the children according to their ages, and the results were compared with those of 25 children of similar age and socio-economic class. The children in the study group were not different from their controls in any of the measured tests.

Leukaemia occurs in approximately one out of 100 000 pregnancies (Caligiuri and Mayer, 1989). Aviles and Niz (1988) examined 17 offspring of patients with acute leukaemia treated during pregnancy. Chemotherapy was given during the pregnancy in each case, including 11 cases during the first trimester. The treatment of acute leukaemia was not modified due to the pregnancy and these patients received at least 80% of the planned dose. Neurological, intellectual and visual-motor-perceptual assessments were performed on the offspring (who ranged in ages between 4 to 22 years), their siblings, and unrelated controls. All children were given the Wechsler or the Bender–Gestalt test according to their age. No differences were detected between the groups on any of the tests. Kozlowski et al (1990) reviewed retrospectively the outcome of first-trimester exposure to low-dose methotrexate in eight patients with rheumatic disease. The duration of treatment ranged from 2 to 20 weeks of gestation. The women were given 7.5 to 10 mg methotrexate weekly. Five full-term live babies were born with a mean follow-up age of 11.5 years (range 3.7–16.7 years). All children reached normal growth and neurodevelopment. None of the children had learning disabilities. One child with speech impairment improved after speech therapy. Unfortunately, the methods of mental assessments were not reported.

The outcome of occupational chemotherapy exposure during pregnancy was addressed by Medkova (1991). The author reported on the pregnancy outcome and neurodevelopment of health personnel's children exposed to small doses of cytostatic medications. Sixty-one children were born to the healthcare workers on the oncology unit. In this group, exposure to antineoplastic agents was proven in five mothers and five fathers. Attention was paid to the children's physical development and possible incidence of dyslexia or dysgraphia. No abnormalities in these areas were found. Formal cognitive tests and statistical comparisons were not reported.

In summary, the data presented regarding late effects of chemotherapy on children's neurodevelopment are incomplete and are hampered by a lack of population-based, well designed studies. It is important to note that the paucity of neurobehavioural and cognitive studies in older children is due to limited late follow-up. The majority of available reports have focused on immediate maternal and fetal pregnancy outcomes, not considering later neurodevelopment as a primary end point, thus using a cross-sectional rather than longitudinal approach. The studies which did address long-term neurodevelopmental aspects typically used retrospective design in order to recruit a sufficient number of cases. Notwithstanding the limitations, these studies are very important as they represent the only existing source of information on the neurodevelopmental outcome of in utero exposure to cytotoxic therapy. The general impression, based on these reports, is that chemotherapy does not have a major impact on later child neurodevelopment. Methodologically, retrospective studies tend to show more adverse outcome than prospective studies, due to reporting bias (e.g.: parents of children with adverse outcome are more likely to report than those with normal outcome). Hence, negative retrospective studies are reassuring. With the increased use of cancer medications in nonmalignant conditions (e.g.: transplant, collagen diseases), it may be possible to recruit larger numbers of children for prospective, longitudinal studies and delineate abnormal outcomes induced by the malignancy itself.

### Glucocorticoids

Glucocorticoids are part of the treatment protocol for many malignancies. They are also used as immunosupressive treatment in organ transplantation and for fetal lung maturation when preterm delivery is suspected. Most comprehensive studies on the neurodevelopmental effects of glucocorticoids were done in late pregnancy. Animal studies indicate that corticosteroids are associated with cognitive and behavioural abnormalities when administered during pregnancy. This has raised concerns about their use in humans (Trautman et al, 1995; Meaney et al, 1982; Angelucci et al, 1985). Table 2 presents the long-term effects of glucocorticoids used in gestation.

A prospective pilot study that investigated the long-term neurodevelopmental effects of prenatal exposure to dexamethasone (DEX) was conducted by Trautman et al (1995). The authors followed 26 children (ranging in age from 6 months to 5.5 years) whose mothers took DEX during pregnancy from weeks 1 to 21. The mothers took DEX for 2–29 weeks for the treatment of fetal risk for congenital adrenal hyperplasia (CAH). The offspring were compared with 14 children who were also at risk for CAH, but had not been exposed to DEX. The authors attempted to separate the effects of maternal disease by controlling for the same medical condition without treatment. The results indicated that DEX-exposed children were less likely to be cognitively delayed on a comprehension-conceptualization measure of development and that DEX-exposed 2- to 3-year-old children ($n$ = 14) displayed more internalizing behaviour than unexposed children of the same age ($n$ = 4). DEX-exposed children also exhibited a tendency to show higher avoidance behaviour and to be more shy and emotional and less sociable than unexposed children. No other significant differences were reported.

Pregnancy outcome and long-term follow-up with DEX used as an immunosuppressive agent taken throughout pregnancy for the treatment of lupus erythematosus (21 children) and heart transplant patients (29 children) were reported (Tincani et al, 1992; Wagoner et al, 1993). The authors indicated that the offspring were doing well at follow-up, but no formal cognitive or behavioural tests were conducted.

The literature on exposure to DEX in late pregnancy suggests that there are no significant cognitive differences between exposed and non-exposed children (MacArthur et al, 1982; Veszelovsky et al., 1981; Collaborative Group on Antenatal Steroid Therapy, 1984). The Collaborative Group on Antenatal Steroid Therapy (1984) was a prospective, randomized, placebo controlled, double-blinded study on the use of antenatal DEX for the prevention of respiratory distress syndrome in infants. The authors evaluated 200 children at 9, 18, and 36 months of age. The Bayley Scales and McCarthy Scales of Children's Abilities were used to assess cognitive and motor development. The results indicated that there were no significant differences in head circumference, cognitive, developmental or neurologic functioning between the placebo and the steroid treatment groups. The authors concluded that there were no detectable effects within the first 3 years of life in children who were exposed antenatally to DEX.

MacArthur et al (1982) conducted a double-blind, controlled study investigating the cognitive and psychosocial development of

 *© 2001 Cancer Research Campaign*

**Table 2** Children exposed in utero to glucocorticoids

| Indication | Author | Time of exposure in pregnancy | Study design | Sample size *n* = 540 | Age assessed | Substance | Duration of substance use | Tests | Results |
|---|---|---|---|---|---|---|---|---|---|
| Lung maturation **in Hungarian | Veszelovszky et al, 1981 | 3rd trimester | Retrospective, controlled | 125 | 12–36 months | Dexamethasone | | Neurological and psychological | No difference from the control group |
| Fetal lung maturation | MacArthur et al, 1981 and 1982 | 3rd trimester | Prospective, controlled | 139 | Age 4 and 6 | Betamethasone | | Stanford-Binet Intelligence Scale, Frostig Visual Perception Test, Vineland Social Maturity Scale, Illinois Test of Psycholinguistic Abilities, Peabody Vocabulary Test, Raven's Matrices, Bender-Gestalt Test | No difference from the control group |
| Fetal lung maturation | Collaborative Group on Antenatal Steroid Therapy (1984) | 3rd trimester | Prospective, randomized, placebo controlled, double-blind | 200 | 36 months | Dexamethasone | | Bayley Scales, McCarthy Scales | No difference from the control group |
| Fetal lung maturation | NIH Consensus Conference, 1995 | 3rd trimester | Analysis of available data | | up to age 12 | Corticosteroids | | | No increased risk of long-term neurodevelopmental impairment |
| Maternal SLE | Tincani et al, 1992 | Throughout pregnancy | Prospective | 21 | 1–85 months | Fluocortolone plus aspirin and azathioprine (if needed) | | No formal tests | No long-term consequences |
| Maternal heart transplants | Wagoner et al, 1993 | During pregnancy | Prospective | 29 | 3 months to 6.5 years | Corticosteroids plus other immunosuppresives | | No formal tests | All children reported to be in good health |
| Fetal congenital adrenal hyperplasia | Trautman et al, 1995 | Started from week 1 to 21 weeks of gestation | Porspective, controlled pilot study | 26 | 6 months to 5.5 years | Dexamethasone | 2–29 weeks | Denver Developmental Questionnaire, Minnesota Child Development Inventory, Child Behaviour Checklist, Temperament Questionnaire, EAS Temperament Survey | More shy, emotional, less sociable, and a trend for greater avoidance than control group. No differences in cognitive abilities. |

© 2001 Cancer Research Campaign children whose mothers were treated antenatally with betamethasone during preterm labour. A total of 250 children, 139 in the betamethasone group and 111 in the control group, were studied through to age 7. The authors used well-established measures of cognitive, academic, and psychosocial development. Their results suggested that there were no significant differences in the cognitive or psychosocial development between the two groups.

The NIH Consensus Conference in 1994 (Anonymous, 1995) summarized data on the effects of corticosteroids for fetal maturation and perinatal outcomes. The results from available studies did not indicate any evidence of adverse long-term outcomes associated with the use of single doses of glucocorticoids for lung maturation in the areas of motor, cognitive, language, memory, concentration, or scholastic achievement skills. There is however evidence that repeated doses, similar to what is encountered in cancer chemotherapy are associated with microcephaly and low birth weight.

The results of a prospective control study by French et al (1999) point to a reduction in head circumference when repeated courses of corticosteroids were given in late pregnancy. Although smaller head circumference may be associated with impaired cognitive outcome, the investigator did not report cognitive impairment.

In conclusion, although the literature on the impact of corticosteroids administration in pregnancy on fetal development is reassuring, more information is required on long-term effects of these substances on the CNS development.

### Bromocriptine

Bromocriptine is an alkaloid that functions as a dopamine receptor agonist suppressing prolactine production and is the treatment of choice for pituitary tumours during pregnancy. Pregnancy outcome and children's functioning in long-term follow-up (up to age 9 years) in hundreds of children exposed in utero to bromocriptine were reported (Turkalj et al, 1982; Konopka et al, 1983; Nader, 1990) with no significant findings. However, no reports of formal tests of cognitive and behavioural functioning are available.

### RADIATION

Radiation is widely used in the diagnosis and treatment of malignancies. Based on live birth rate statistics in the US, about 33 000 women each year are exposed to diagnostic abdominal radiation in early pregnancy (US Department of Health and Human Services, 1976). The developing embryo and fetus are extremely sensitive to ionizing radiation (Brent, 1980). Fetal structures exhibit various susceptibilities to ionizing radiation and the human brain seems to be the most sensitive organ. The CNS maintains its sensitivity to radiation throughout gestation and into the neonatal period, when morphological changes can still be observed. Ionizing radiation is a CNS teratogen and is a recognized cause of mental retardation. It has been suggested to be a more serious fetal risk than maternal cancer following in utero exposure (Hoel, 1987). The lowest dose of radiation that produces significant behavioural changes postnatally at any gestational day is 0.2 Gy (Schull et al, 1990).

Although radiation is one of the most studied environmental hazards in animal species, there are very few human studies available. Brent (1980) hypothesized that a radiation dose as low as 50 rad (0.5 Gy) in humans may be harmful to the fetus, especially to its CNS. Dekaban (1968), in a retrospective analysis, reported that 22 infants who were exposed to radiation in the third to twentieth week of gestation for treatment purposes, were microcephalic and/or mentally retarded. Woo et al (1992) reported results of 16 women treated for nodular sclerosing Hodgkin's disease. Seven women were treated during the first trimester, 10 in the second, and 8 in the third. Sixteen women received 35–40 Gy for supradiaphragmatic nodes. The uterus was shielded by a 4 to 5 hold-value layer of lead. The estimated dose to the mid-fetus ranged from 1.4 to 5.5 cGY for 6 MV photons and 10 to 13.6 cGY for cobalt 60. All 16 patients subsequently delivered full-term normal infants, who, at the time of assessment, were found to be physically and mentally normal. Formal cognitive tests were not reported.

The experience at Hiroshima and Nagasaki is the most commonly sited source of knowledge considering long-term effects of radiation on the human embryo and fetus. These population based studies addressed relationships between dose of radiation and gestational age. Analysis of the data of survivors of the atomic bombings in Japan using refined estimates of the absorbed fetal dose demonstrated that the highest risk of brain damage occurred at 8–15 weeks of gestational age (Otake and Schull, 1984). These data were consistent with a linear dose-response model, which did not indicate the existence of a threshold level.

In contrast, the data collected for in utero exposures after the fifteenth week of gestation were not linearly related to dose, suggesting that a nonlinear model with a threshold dose for radiation effects best fits the data for this period of gestation. Radiation exposure before the eighth week of gestation and after the twenty-fifth week was not associated with an increased risk of mental retardation. Yoshimaru et al (1991) assessed school performance of prenatally exposed survivors of the atomic bombings using the DS86 dosimetry system and found that damage to the fetus exposed at 16–25 weeks after fertilization appeared similar to that seen in the 8–15 week group. Other studies of this population also suggest that radiation exposure in utero may affect intelligence test scores, with the greatest sensitivity during the eighth to fifteenth week of gestation (Mettler et al, 1985). One estimate of the dose-response relationship was a 20-point loss of IQ for each additional 1 Gy of exposure. The relationship between dose and intelligence test scores is not yet well-established, and the findings have to be refined to a demonstrable level of statistical significance or clinical relevance (Mettler et al, 1985). Jensh and Brent (1987) demonstrated a downward shift in the Gaussian distribution of IQ with an estimated probability coefficient indicating a loss of 30 IQ points per 1 Gy fetal dose at 8–15 weeks after conception. A similar, but smaller shift to lower intelligence was detectable following exposure at 6–25 weeks of gestation, but not at other periods of pregnancy. Several reports did not find mental retardation in 19 children exposed to 0.015 and 0.1 Gy and among 1458 children exposed to low diagnostic doses of radiation (Meyer and Tonascia, 1981). However, behavioural tests were not performed and these results should be interpreted with caution because of methodological limitations of the study design. Conversely, in a case control study, Granoth (1979) assessed the association of diagnostic X-ray examinations with the occurrence of CNS defects and found a significant increase in anencephaly, hydrocephaly, and microcephaly when the study group is compared with matched control infants. The cognitive and behavioural effects of low-dose prenatal diagnostic radiation has not been elucidated. As well, it is unknown whether there are behavioural sequelae due to exposure to radiation in the area of the electromagnetic spectrum. To date it is believed that fetal exposure to less than 0.05 Gy does not increase the teratogenic risk (Jensh and Brent, 1986).

 *© 2001 Cancer Research Campaign*

In summary, ionizing radiation fulfills all of Wilson's criteria for a teratogen (Vorhees, 1986). Being neurotropic, radiation is capable of producing behavioural teratogenic effects that are demonstrable at doses below those causing obvious structural malformations (Vorhees, 1986).

In addition to the classic triad produced by a teratogenic substance, ionizing radiation was observed to cause obvious behavioural alternations in animals. As suggested by Jensh and Brent (1986) the inseparable radiation teratogenic effect on behaviour must be added to the triad thus creating a 'tetrad'.

### CONCLUSION

Malignancy poses a difficult challenge to both patients and physicians, creating a conflict between optimal maternal therapy and fetal well-being. However, most malignant conditions are not absolute contraindications for pregnancy. Unfortunately, neurodevelopmental and behavioural effects, both important parts of the teratogenic spectrum regarding drug or treatment safety, have been only sparsely addressed. Mental health effects, which act as strong predictors of a child's quality of life, merit closer attention, as does the cognitive and behavioural effects of these drugs. At the present time little is known on the long term mental health effects in young children exposed in utero to treatment for maternal cancer. However, existing studies on cancer chemotherapy beyond the first trimester have failed to show neurotoxicity. In contrast, repeated doses of corticosteroids and radiation are definitely teratogenic. Further research is required to assess the behavioural teratology of cancer and its management.

### REFERENCES

Angelucci L, Patacchioli FR, Scaccianoce S, Di Sciull A, Maccari S and Cardillo A (1985) A model for later-life effects of perinatal drug exposure: Maternal hormone mediation. *Neurobehav Toxicol Teratol* **7**: 511–517

Anonymous (1995) Effect of corticosteroids for fetal maturation on perinatal outcomes. NIH Consensus Development Panel on the Effect of Corticosteroids for Fetal Maturation on Perinatal Outcomes. *JAMA* **273**: 413–418

Aviles A and Niz J (1988) Long-term follow-up of children born to mothers with acute leukemia during pregnancy. *Med Pediatr Oncol* **16**: 3–6

Aviles A, Zepeda G and Cruz J (1991) Hodgkin's disease during pregnancy. Study of late effects in the newborn. *Bol Med Hosp Infant Mex* **48**: 622–626

Baisogolov GD and Shishkin IP (1985) Course of pregnancy and condition of infants born to patients treated for lymphogranulomatosis [Russian]. *Med Radiol (Mosk)* **30**: 35–37

Balcewicz-Sablinska K, Ciesluk S, Kopec I, Slomkowski M and Maj S (1990) Analysis of pregnancy, labor, child development and disease course in women with Hodgkin's disease. *Acta Haematol Pol* **21**: 72–80

Blatt J, Mulvihill JJ, Ziegler JL, Yong RC and Poplack DG (1980) Pregnancy outcome following cancer chemotherapy. *Am J Med* **69**: 828–832

Brent RL (1980) Radiation teratogenesis. *Teratology* **21**: 281–298

Brown JE, Jacobson HN, Askue LH and Peick MG (1981) Influence of pregnancy weight gain on the size of infants born to underweight women. *Obstet Gynecol* **57**: 13–17

Caligiuri MA and Mayer RJ (1989) Pregnancy and Leukemia. *Semin Oncol* **16**: 388–396

Cantini E and Yanes B (1984) Acute myelogenous leukemia in pregnancy. *South Med J* **77**: 1050–1052

Chambers CD, Johnson KA, Dick LM, Felix RJ and Jones KL (1998) Maternal fever and birth outcome: a prospective study. *Teratology* **58**: 251–257

Clarren SK, Smith DW, Harvey MA and Ward KH (1979) Hyperthermia: a prospective evaluation of a possible teratogenic agent in man. *J Pediatr* **95**: 81–83

Collaborative Group on Antenatal Steroid Therapy (1984) Effects of antenatal dexamethasone administration in the infant: long-term follow-up. *J Pediatr* **104**: 259–267

Dekaban AS (1968) Abnormalities in children exposed to x-radiation during various stages of gestation: tentative timetable of radiation injury to the human fetus. *J Nucl Med* **9**: 412–477

Doll DC, Ringenberg QS and Yarbo JW (1988) Management of cancer during pregnancy. *Arch Intern Med* **148**: 2058–2064

French NP, Hagan R, Evans SF, Godfrey MRN and Newnham J (1999) Repeated antenatal corticosteroids: Size at birth and subsequent development. *Am J Obstet Gynecol* **180**: 114–121

Friedman JM (1988) Teratogen update: anesthetic agents. *Teratology* **37**: 69–77

Gililland J and Weinstein L (1983) The effects of cancer chemotherapeutic agents on the developing fetus. *Obstet Gynecol Surv* **38**: 6–13

Granoth G (1979) Defects of the central nervous system in Finland. IV Associations with diagnostic X-ray examinations. *Am J Obstet Gynecol* **133**: 191–194

Harada M (1978) Congenital Minamata disease: intrauterine methylmercury poisoning. *Teratology* **18**: 285–288

Hoel DG (1987) Radiation risk estimation models. *Environ Health Perspect* **75**: 105–107

Jensh RP and Brent RL (1986) Effects of 0.6-Gy prenatal X irradiation on postnatal neurophysiologic development in the Wistar rat. *Proc Soc Exp Biol Med* **181**: 611–619

Jensh RP and Brent RL (1987) The effect of low level prenatal X-irradiation on postnatal development in the Wistar rat. *Proc Soc Exp Biol Med* **184**: 256–263

Kallen B and Mazze RI (1990) Neural tube defects and first trimester operations. *Teratology* **41**: 717–720

Konopka P, Raymond JP, Merceron RE and Seneze J (1983) Continuous administration of bromocriptine in the prevention of neurological complications in pregnant women with prolactinomas. *Am J Obstet Gynecol* **146**: 935–938

Koren G and Nulman I (1994) Teratogenic Drugs and Chemicals in Humans. In: *Maternal Fetal Toxicology: Clinician's Guide*, 2nd edn, Koren G (ed.) pp 33–48. Marcel Dekker: New York

Kozlowski RD, Steinbrunner JV and MacKenzie AH (1990) Outcome of first-trimester exposure to low-dose methotrexate in eight patients with rheumatic disease. *Am J Med* **88**: 589–592

Layde PM, Edmonds LD and Erickson JD (1980) Maternal fever and neural tube defects. *Teratology* **21**: 105–108

MacArthur BA, Howie RN, Dezoete JA and Elkins J (1982) School progress and cognitive development of 6-year old children whose mothers were treated antenatally with betamethasone. *Pediatrics* **70**: 99–105

Meaney MJ, Stewart J and Beatty WW (1982) The influence of glucocorticoids during the neonatal period on the development of play-fighting in Norway rat pups. *Horm Behav* **16**: 475–491

Medkova J (1991) Analysis of the health condition of the children born to the personnel exposed to cytostatics at an oncology unit. *Acta Univ Palacki Olomuc Fac Med* **130**: 323–332

Metcoff J, Costiloe JP, Crosby W, Bentle L, Seshachalam D, Sandstead HH, Bodwell CE, Weaver F and McClain P (1981) Maternal nutrition and fetal outcome. *Am J Clin Nutr* **34**: 708–721 (Suppl 4)

Mettler FA and Moseley RD (1985) *Medical Effects of Ionizing Radiation* pp 206–209. Grune & Stratton: New York

Meyer MB and Tonascia J (1981) Long term effects of prenatal x-ray of human females. *Am J Epidemiol* **114**: 317–326

Miller P, Smith DW and Shepard TH (1978) Maternal hyperthermia as a possible cause for anencephaly. *Lancet* **1**: 519–521

Nader S (1990) Pituitary disorders and pregnancy. *Semin Perinatol* **14**: 24–33

Naeye RL and Chez RA (1981) Effects of maternal acetonuria and low pregnancy weight gain on children's psychomotor development. *Am J Obstet Gynecol* **139**: 189–193

Odom LD, Plouffe L and Butler WJ (1990) 5-Fluorouracil exposure during the period of conception: Report on two cases. *Am J Obstet Gynecol* **163**: 76–77

Otake M and Schull WJ (1984) In utero exposure to A-bomb radiation and mental retardation: a reassessment. *Br J Radiol* **57**: 409–414

Potter JF and Schoeneman M (1970) Metastasis of maternal cancer to the placenta and fetus. *Cancer* **25**: 380–388

Pritchard JA, MacDonald PC and Gant NF (1985) *Williams Obstetrics*, 17th edn. Appleton-Century-Crofts: Norwalk, CT

Reynoso EE, Sheperd FA, Messner HA, Farquharson HA, Garvey MB and Baker MA (1987) Acute leukemia during pregnancy: the Toronto Leukemia Study group experience with long-term follow-up of children exposed in utero to chemotherapeutic agents. *J Clin Oncol* **5**: 1098–1106

Schardein JL (1985) Cancer chemotherapeutic agents. In: *Chemically Induced Birth Defects*, Schardein JL (ed.) pp 467. Marcel Dekker: NY and Basel

Schull WJ, Norton S and Jensh RP (1990) Ionizing radiation and the developing brain. *Neurotoxicol Teratol* **12**: 249–260

*© 2001 Cancer Research Campaign*

Stein Z, Susser M, Saenger G and Marolla F (1972) Nutrition and mental performance. *Science* **178**: 708–713

Sylvester GC, Khoury MJ, Lu X and Erickson D (1994) First-trimester anesthesia exposure and the risk of central nervous system defects: A population-based case-control study. *Am J Public Health* **84**: 1757–1760

Tincani A, Faden D, Tarantini M, Lojacono A, Tanzi P, Gastaldi A, Di Mario C, Spatola L, Cattaneo R and Balestrieri G (1992) SLE and pregnancy: a prospective study. *Clin Exp Rheumatol* **10**: 439–446

Trautman PD, Meyer-Bahlburg HF, Postelnek J and New MI (1995) Effects of early prenatal dexamethasone on the cognitive and behavioral development of young children: results of a pilot study. *Psychoneuroendocrinology* **20**: 439–449

Turkalj I, Braun P and Krupp P (1982) Surveillance of bromocriptine in pregnancy. *JAMA* **247**: 1589–1591

US Department of Health and Human Services: Vital Statistics of the Unites Stat, 1976. Bol. Natality. Hyattsville, MD, National Center for Health Statistics, 1980

Veszelovszky I, Farkasinszky T, Nogy J, Bodis L and Szilard J (1981) Psychological and neurosomatic follow-up studies of children of mother treated with dexamethasone. *Orv Hetil* **122**: 629–631

Vorhees CV (1986) Principles of behavioural teratology. In: *Handbook of Behavioural Teratology*, Riley EP and Vorhees CV (eds) pp 23–48. Plenum Press: New York

Wagoner LE, Taylor DO, Olsen SL, Price GD, Rasmussen LG, Larsen CB, Scott JR and Renlund DG (1994) Immunosuppressive therapy, management, and outcome of heart transplant recipients during pregnancy. *J Heart Lung Transplant* **12**: 993–1000

Webster WS, Lipson AH and Sulik KK (1988) Interference with gastrulation during the third week of pregnancy as a cause of some facial abnormalities and CNS defects. *Am J Med Genet* **31**: 505–512

Woo SY, Fuller LM, Cundiff JH, Bondy ML, Hagemeister FB, McLanghlin P, Velasquez WS, Swan F, Rodriguez MA and Cabanillas F (1992) Radiotherapy during pregnancy for clinical stages IA-IIA Hodgkin's disease. *Int J Radiat Oncol Biol Phys* **23**: 407–412

Yoshimaru H, Otake M, Fujikoshi Y and Schull WJ (1991) Effect on school performance of prenatal exposure to Hiroshima atomic bomb. *Nippon Eiseigaku Zasshi* **46**: 747–754

*© 2001 Cancer Research Campaign*
